# Piloting a computer assisted telephone interview: the FUCHSIA Women’s Study

**DOI:** 10.1186/s12905-014-0149-y

**Published:** 2014-11-30

**Authors:** Helen B Chin, Candice Y Johnson, Konny H Kim, Jessica H Knight, Ann C Mertens, Pamela J Mink, Regina M Simeone, Jill J Woodard, Penelope P Howards

**Affiliations:** Department of Epidemiology, Rollins School of Public Health, Emory University, 1518 Clifton Road NE, CNR 3rd Floor, Atlanta, GA 30322 USA; Aflac Cancer Center, Department of Pediatrics, Emory University School of Medicine, 2015 Uppergate Road, Atlanta, GA 30322 USA; Division of Applied Research, Allina Health, Mail Route 10039, 2925 Chicago Ave S, Minneapolis, MN 55407 USA

**Keywords:** Pilot testing, Telephone interview, Recruitment, Survivorship, Cancer, Fertility

## Abstract

**Background:**

Loss of fertility has been reported as an important concern of reproductive age women diagnosed with cancer. The Furthering Understanding of Cancer, Health, and Survivorship In Adult (FUCHSIA) Women’s Study examines how cancer treatment affects the fertility of cancer survivors who were diagnosed during their reproductive years. In this paper we discuss the process of developing and pilot testing the FUCHSIA computer assisted telephone interview (CATI).

**Methods:**

The CATI was developed in several phases and pilot tested twice to evaluate several aspects of the instrument including question sequencing, understandability of the questions, and women’s comfort with certain questions. Participants were recruited from cancer and infertility support groups and study team contacts.

**Results:**

Fifty-two women were recruited and participated in the first pilot. The participants had a mean age of 31.5 years, 17.3% had cancer, and 38.5% experienced a period of infertility. Twenty-four women participated in the second pilot with similar representation.

**Conclusions:**

The collection of detailed information on reproductive outcomes with the CATI may improve the understanding of how cancer treatment during the reproductive years affects female fertility. The pilot studies provided important information to improve the CATI before the full study. Our comprehensive recruitment strategy allowed us to interview a diverse group of women to ensure that questions and answer choices were easily interpreted, check complicated skip patterns and the flow of questions, and evaluate the length of the interview. This experience can be used to help inform others in what steps can be useful for developing telephone interviews for research studies.

**Electronic supplementary material:**

The online version of this article (doi:10.1186/s12905-014-0149-y) contains supplementary material, which is available to authorized users.

## Background

Young adult cancer survivors represent a large, but understudied population compared to older adult and pediatric cancer populations. In the past decade, the National Cancer Institute (NCI) issued a report emphasizing the need for more research on individuals diagnosed with adolescent and young adult cancers [1]. One of the challenges of studying survivorship issues among young adult cancer survivors is that the population is heterogeneous with respect to the types of cancers they experience, the treatments they receive, as well as sociocultural factors such as insurance status and familial situation, which ranges from being dependent on their parents to being independent to being married [1,2]. Further, unlike pediatric cancer patients, young adult cancer patients are less likely to be treated at cancer centers; their care is usually community based [1,3,4].

To study such a diverse population, self-reported information in the form of questionnaires is critical to further these needed research initiatives. Pilot testing is an important step in the development of questionnaires for health research [5]. These tests serve as rehearsals for the full study, helping researchers evaluate the quality of data collected from the interview and identify questions that may be difficult for participants to answer [5,6]. Although it is critical to resolve problems with a health interview before conducting the larger study, testing an interview can be resource intensive and detailed descriptions of this pilot testing process are uncommon in the literature [7,8]. Reports of issues and achievements that occur during the pilot phase would thus be useful to researchers planning studies using similar methods [7]. In this paper, we describe the challenges faced and lessons learned while piloting a computer-assisted telephone interview (CATI) for a population-based study of women’s health in survivors of young adult cancers.

Increasingly, it has been recognized that certain life-saving cancer treatments have gonadotoxic effects in cancer survivors [9-12]. Loss of fertility has been reported to be almost as important to reproductive aged women diagnosed with cancer as concerns about survival [13,14]. The Furthering Understanding of Cancer, Health, and Survivorship In Adult (FUCHSIA) Women’s Study examines how cancer treatment affects the fertility of cancer survivors who were diagnosed during their reproductive years (age 20-35 years). We developed a CATI to collect detailed health information from female cancer survivors who were at least 2 years post diagnosis, but still of reproductive age (22– 45 years), as well as comparison women of the same age who have never been diagnosed with cancer. Prior to using the CATI in the main study, we piloted it to evaluate several aspects of the instrument including question sequencing, understandability of the questions, and women’s willingness to answer certain questions. In this paper we discuss the process by which the initial CATI was developed, and how the piloting process was used to identify and address specific areas of concern.

## Methods

### Initial CATI development

**Figure 1:**
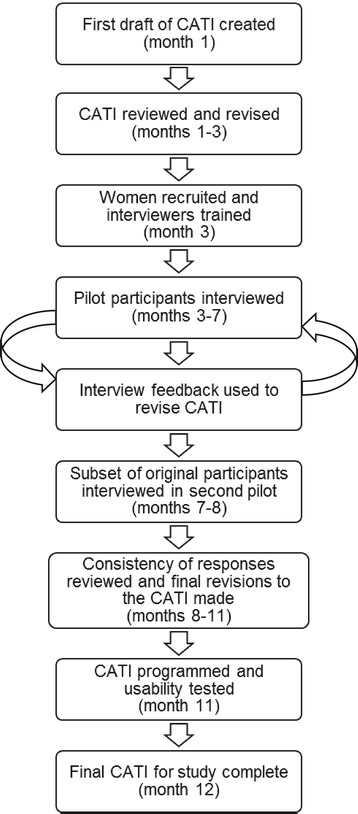
**Timeline of CATI development and piloting.**

The FUCHSIA Women’s Study CATI was developed in several phases over 12 months (Figure 1). First, we identified the major fertility-related factors to be covered by the CATI. Existing published and unpublished survey instruments were evaluated to determine whether they included questions that addressed the topics of interest and were suitable for a telephone interview. These instruments were collected in several ways. We conducted literature reviews to find relevant studies that identified a specific interview or questionnaire. These data collection tools were then retrieved through internet searches or contacting the principal investigators of the study. Survey instruments from unpublished studies were obtained through communications with colleagues. Instruments for 25 published and unpublished studies were used to inform CATI development (see Additional file 1). The first draft of the CATI was comprised of a combination of questions adopted directly from other instruments, modified questions based on other instruments, and new questions to address specific aims of the study.

The study team, which included members with expertise in reproductive epidemiology, cancer epidemiology, epidemiologic methods, and clinical medicine, reviewed the first draft of the CATI. The primary goals at this stage were to: 1) ensure the questions were capturing information that would address gaps in the literature, 2) ensure completeness of the information collected, and 3) identify questions that could be removed to decrease the length of the interview. Revisions to the CATI were evaluated through mock interviews based on pre-specified reproductive histories. After internal review was complete, the CATI was sent to content and methods subject matter experts for further review. The resulting revision of the CATI was used in the pilot study.

### Pilot Study 1

The goals of the first pilot study were to: 1) ensure that the questions were easily interpreted by the participants, 2) ensure that the questions and answer options were appropriate for a wide range of experiences, 3) confirm that no questions were considered offensive, inappropriate, or exclusive, 4) thoroughly check the complicated skip patterns and flow of the questions, and 5) evaluate the length of the CATI. The pilot study was approved by the Emory Institutional Review Board.

To be eligible for the pilot, participants had to be 20-50 years old. A broader eligible age range was used for the pilot study compared with the full study to maximize recruitment. Our a priori goal was to test the interview with 30 participants. To accomplish this, a convenience sample was recruited from three sources: 1) cancer survivor support groups, 2) infertility support groups, and 3) personal and professional contacts of the study team. Support groups were identified through internet searches to find women treated for cancer and women who experienced infertility. Support groups in the state of Georgia were excluded to minimize overlap with any of the women targeted for recruitment in the full study. We sent emails to 123 infertility support groups and 50 cancer support groups. Support group leaders were asked to send the information about the pilot study to their group members. We supplemented the participants from support groups with our own contacts to ensure that our questions could capture experiences that might be underrepresented. We sent emails to 56 contacts of study team members. Specifically, we reached out to single women who used artificial reproductive technology (ART), women who adopted children, women with female partners, and women early in their reproductive years. Further, women with no known cancer or infertility were used to test the CATI as it might be applied to the comparison group women who never had cancer treatments. All women were encouraged to forward the official pilot email to eligible family and friends who might also be interested in the study.

We recruited 52 women with approximately 20% from an infertility support group, 10% from a cancer support group, and the remaining women from personal and professional contacts (Table 1). The participants had a mean age of 31.5 years, 17.3% had cancer, and 38.5% experienced periods of infertility, which we defined as regular unprotected sex for 6 months or longer without a pregnancy.

**Table 1:** **Women participating in pilots by source**

**Source** ^**a**^	**First pilot N (% of total participants)**	**Second pilot N (% of total participants)**
Infertility group^b^	10 (19.2)	5 (20.8)
Cancer group^c^	5 (9.6)	3 (12.5)
Study team contacts (total)	36 (69.2)	15 (62.5)
Fertility problems	4	3
Cancer	3	1
Both fertility and cancer problems	1	0
No known cancer or fertility problems	28	11
Total	52	24

^a^1 woman with unknown source participated in both the first and second pilot.

^b^1 woman recruited from an infertility group also was a cancer survivor.

^c^3 women recruited from a cancer group also experienced infertility.

All pilot interviews were conducted after receiving consent over the phone by trained study staff. For the pilot, we used a paper copy of the CATI questions so that interviewers could take notes next to any question or section where an issue arose. At the end of the interview, women were asked a set of additional open-ended questions regarding their interview experience. After the interview was complete, interviewers recorded their impressions of the interview.

Information such as how long the CATI took to administer, the flow of skip patterns, and participant and interviewer feedback were used to make revisions to the CATI. The most current version of the CATI was used for each interview to avoid problems encountered in previous versions. Revisions to the CATI continued after completing the first round of pilot interviews.

### Pilot Study 2

Three months after the initial pilot study, we re-contacted all eligible participants via email to invite them to participate in a pilot of the revised CATI. Twenty-four women agreed to participate in this second pilot study (Table 1). All of the women participating in the second pilot study were re-consented and interviewed using the version of the CATI that incorporated all revisions from the first pilot. We had 2 objectives for the second pilot study: 1) to check the consistency of participants’ responses to unchanged questions, and 2) to ensure that the revised questionnaire was easier to understand. While we did not provide an incentive for women who participated in the first pilot alone, women who agreed to participate in the second pilot received a $10 gift card to thank them for their continued participation. The CATI was revised again at the end of the second pilot study.

### CATI programming and usability test

Westat, a private research corporation, was contracted to program the CATI into interactive software, administer a usability test, and perform interviews for the full study. The study staff at Emory tested the CATI programming by entering data collected during the pilot studies into the Westat version of the CATI. During this process, the team was able to identify missing or mistyped questions as well as problems with skip patterns. After the programmed version of the CATI was finalized, Westat administered a usability test of the CATI to a convenience sample of 13 diverse women. Women who contacted the study to participate in the pilot after recruitment was completed and a sample of comparison women eligible for the main study were recruited. Women who had experienced infertility or survived cancer were prioritized. The usability test was done to test the programming of the CATI and evaluate how Westat interviewers would administer the CATI to women in the full study. The Westat interviewers provided feedback from the usability testing on their experiences with the instrument. After the usability test, we finalized the CATI.

## Results

Pilot and usability testing allowed the study team to address 5 critical issues that helped improve the CATI before it was administered to eligible FUCHSIA participants.

### Interpretation

To assess if study participants’ interpretation of questions asked of them was consistent with the way investigators intended, we evaluated the CATI in two ways. First, women in the pilot interviews were made aware of the purpose of the study and encouraged to provide feedback during the interview. Second, women were asked about their interpretation of questions that were identified as potentially problematic and alternative wording was discussed. The study staff monitored questions that were considered unclear for future revisions.

As an example, one question that was difficult for participants to answer as intended by the investigators referred to periods of infertility (Table 2). The purpose of this question was to ascertain times women experienced an infertile period regardless of whether or not they were trying to get pregnant at the time. One problem was that women who never had sexual intercourse with a male partner did not know how to answer the original question. This was addressed by adding a screening question for ever having intercourse with a male partner. Another problem was that some women did not consider time when they were not attempting pregnancy. The study team addressed this by asking separate questions by pregnancy intention. However, participant and interviewer feedback indicated that there was confusion about the difference between the two questions. Rearranging the information in the original question and inserting pauses improved participants’ understanding.

**Table 2:** **Development of the infertility question**

Original question^a,c^	• “Have you ever had a period of 12 months when you did not get pregnant even though you were having regular sexual intercourse and not doing anything to prevent pregnancy? Include times when you weren’t trying to get pregnant. Regular intercourse includes having sex at least 3 times per month.”
Intermediate questions^b,c^	• “Have you ever had sexual intercourse with a male partner?”
	• “Have you ever tried to get pregnant for 6 months or more? Only count months when you had unprotected sex with a man at least 3 times per month with the intention of getting pregnant.”
	• “Now, think about times you were not trying to become pregnant. Was there ever a period of 6 months or longer when you were having regular intercourse with a man and doing nothing to prevent pregnancy but you did not become pregnant? Regular intercourse means at least 3 times per month.”
Final question^c^	• “Have you ever had sexual intercourse with a male partner?”
	• “Has there ever been a period of time during which you had unprotected sex with a male partner for 6 months or longer but you did not get pregnant? Only count periods of time when you had sex at least 3 times a month.”

^a^Originally the question asked about a 12 month period of time, but was changed to a 6 month period of time to capture periods of subfertility and not just infertility, defined as the failure to conceive after 1 year of trying.

^b^One example of several intermediate questions.

^c^Questions looped to capture multiple periods of infertility.

### Acceptable level of detail

To accurately answer research questions it is important to request information that is as detailed as possible without being too specific for the interviewee to remember. One of the most detailed sections of the CATI asked about information on fertility treatment. This section included questions on the number of embryos frozen, stage at implantation, and whether fresh or frozen embryos were used for each cycle of treatment. In general, participants in the pilot studies were able to report this detailed information. However, we recognize many participants were members of infertility support groups and might be more aware of their treatments. Nevertheless, some of the participants who were not in support groups also reported being comfortable answering these questions.

One exception was among women who had their eggs retrieved and embryos frozen multiple times. Originally, the interview asked women which egg retrieval cycle was used to fertilize the frozen embryos that were being used for each attempted pregnancy. Women had difficulty identifying which egg retrieval cycle their frozen embryos originated from, but did know whether they had fresh or frozen embryos implanted. Using this information, we resolved the problem by splitting the question. First they were asked if they froze any embryos at each round of treatment. Second, if they had embryos implanted, they were asked if they were fresh or frozen. This change eliminated the problem and still provided sufficient information about the use of fresh and frozen embryos.

### Range of experiences

To test the parts of a study interview that were addressing the main goals of a study, it was necessary to recruit participants with a wide range of experiences. In this study, our main outcomes are fertility related, so we recruited women from infertility support groups as well as contacts who used ART for various reasons (e.g., male partner infertility, female partner, single parent). Eighteen women interviewed had personal experiences with infertility and their medical histories helped test the fertility-related sections of the interview. These women were able to give us feedback on areas where we needed to revise the wording of our questions to include their experiences. The women with no known problems with fertility still provided feedback on the menstrual cycle and pregnancy sections, which were a source of key outcomes for the full study. This was important because the questions in the CATI were not open-ended response. One example was creating the answer choices for the question “When you went to see a doctor for help becoming pregnant, what was your primary reason for seeking help?” The final CATI included 17 choices for this question including, “single, and want to have a child”, “in a relationship with a female”, and “partner had a vasectomy”.

### Length

When developing a study interview, there is a balance between comprehensiveness and participant burden. Our original goal was an interview that could be completed in 35 minutes. During the pilot, we found that the CATI took approximately 40 minutes overall, but longer if the woman had received cancer and/or fertility treatments and shorter for women who had less complex medical histories. Fortunately, we also learned that the topics covered by the CATI were ones that were important to most women, including those who were not part of support groups, and therefore, they were willing to commit the time to participate in the interview, even without an incentive. Although the infertility and pregnancy sections were two of the longest in the interview, several women expressed appreciation that those questions were included.

### Consistency

A goal of any study interview is that questions can be answered with consistency. By comparing responses given by the same women in the first and second pilot studies, we were able to evaluate whether women answered questions consistently. Of particular interest was the age women reported different events in their lives because we dated each life event reported by the participant’s age. For main events, such as age at the birth of first child and age at first occurrence of infertility, participants were able to consistently report these ages within 1 year.

## Discussion

There is a paucity of literature available to aid researchers in the design and piloting of questionnaires for research. For our study, the CATI pilot studies were critical to the development of a study instrument that would be acceptable and understandable to participants and would elicit informative data for the FUCHSIA Women’s Study. Overall recruitment was successful. In general, women were interested in participating in the pilot because they had experienced or knew someone who had experienced cancer or infertility. Our comprehensive recruitment strategy allowed us to interview a diverse group of women and achieve the goals of the pilot test. This method can also be used for targeting other groups for recruitment.

Challenges of developing a study interview include interpretation of the questions, providing an acceptable level of detail to the researchers, inclusiveness of a range of life experiences, acceptable length, and the ability of participants to answer questions consistently. The pilot enabled us to address these challenges and to identify which questions were problematic and which questions were not. One of the problems identified was question and answer choices that did not make sense to women with less common experiences. Another problem was with the phrasing of detailed questions in the CATI that was sometimes difficult for women to understand. Participants were also essential in testing the skip patterns and interview flow. They also provided information on how long the interview would take to complete for each of our target groups. One telephone survey methods text states that respondents will suffer from interview fatigue after 20-30 minutes of time answering questions on the telephone [15]. The CATI took approximately twice as long to complete among women with more complicated medical histories. Participants of the pilot study provided useful feedback in terms of the acceptable length of the different sections of the CATI as well as the overall length.

There are few published studies on conducting a pilot test of a telephone interview. Literature on pilot studies of telephone interviews are mostly focused on sampling techniques, comparing the quality of information collected by telephone to face-to-face interviews, or discussing the feasibility of conducting a full scale study [16-18]. Although these issues in study design are important, the issues raised in this pilot paper provide a more detailed description of the process of developing, recruiting participants, and pilot testing a telephone interview. The steps taken in these three phases of development of the CATI were purposefully conducted to maximize the value of the information gathered in the pilot to improve the study instrument.

The CATI development and piloting process had many strengths, but also some limitations. The piloting process helped to test the logic and flow of the study questions, but did not formally test question validity. Our recruitment procedures, while useful in recruiting women with diverse backgrounds to test the questions in various skip patterns in the CATI, was unlike the recruitment procedures to be used in the full study. Therefore, the sampling strategy in the pilot test was not informative about sampling for the full study. Another limitation is that while recruitment for the pilot study allowed us to test the interview with women who had experienced our outcomes of interest, they may have had better recall of fertility-related events than the population that would be recruited for the full study. Because many of our outcomes were rare, we would not have been able to test all of the sections of the CATI without using this type of recruitment method.

## Conclusions

The benefits of pilot testing the CATI were multifold. First, it was useful in determining women’s willingness to participate in a lengthy interview, allowing us to collect more detailed information than if we adhered to traditional guidelines. Second, it confirmed that women would be open to answering questions on certain sensitive health topics. Third, the pilot interviews allowed for the collection of some preliminary data that was used to inform later revisions. Fourth, the process allowed for the identification of problems that needed to be resolved but also identified situations where things were better than expected. In terms of successful questions, we learned from the pilot that women could answer detailed questions about their fertility treatments in a telephone interview format. We also learned that women appreciated being asked certain questions, such as their desire for children. These successes were just as important to building the final CATI as the problems identified. Our experience with this pilot study provided important information needed to improve the CATI before it went into the field. This experience can be used to help inform others in what steps can be useful for developing telephone interviews for research studies.

## Additional file

Additional file 1:
**Appendix A.** Instruments for 25 published and unpublished studies used to develop the computer assisted telephone interview (CATI) for the Furthering Understanding of Cancer, Health, and Survivorship in Adult (FUCHSIA) Women’s Study.
