# Supplementary material for: Piloting a computer assisted telephone interview: the FUCHSIA Women’s Study
Source: BMC Womens Health. 2014 Nov 30;14:149. doi: 10.1186/s12905-014-0149-y (PMC4261975; doi:10.1186/s12905-014-0149-y)
Supplement: Additional file 1: — Appendix A. Instruments for 25 published and unpublished studies used to develop the computer assisted telephone interview (CATI) for the Furthering Understanding of Cancer, Health, and Survivorship in Adult (FUCHSIA) Women’s Study. [file 12905_2014_149_MOESM1_ESM.doc]

**Appendix A: Instruments for 25 published and unpublished studies reviewed to develop the computer assisted telephone interview (CATI) for the Furthering Understanding of Cancer, Health, and Survivorship in Adult (FUCHSIA) Women’s Study** A link to the study instrument when publicly available or reference to a published paper describing the instrument are provided.

1. Agricultural Health Study/Michael Alvanja, DrPH/National Cancer Institute (<http://aghealth.nih.gov/collaboration/questionnaires.html>)
2. Australian Fertility Decision Making Project/Ruth Weston, PSM/Australian Institute of Family Studies (<http://www.aifs.gov.au/institute/pubs/resreport11/main.html>)
3. BioCycle/Enrique F. Schisterman, PhD/National Institute of Child Health and Human Development
4. Cancer And Menopause Study/Patricia A. Ganz, MD/University of California Los Angeles (Ganz PA, Greendale GA, Petersen L, Kahn B, Bower JE. Breast Cancer in Younger Women:  Reproductive and Late Health Effects of Treatment. Journal of Clinical Oncology. 2003 Nov 15;21(22):4184-93.)
5. DES Daughters Reproductive Health Study/Allen Wilcox, MD, PhD/National Institute of Environmental Health Sciences (<http://www.niehs.nih.gov/research/atniehs/labs/assets/docs/a_d/des_daughters_508.pdf>)
6. Early Pregnancy Study/Allen Wilcox, MD, PhD/National Institute of Environmental Health Sciences (<http://www.niehs.nih.gov/research/atniehs/labs/epi/studies/eps/question/index.cfm>)
7. Effects of the World Trade Center Disaster on Pregnant Women and Their Infants/Gertrud Berkowitz, PhD/Mount Sinai School of Medicine
8. ENDO/Germaine Buck Louis, PhD/National Institute of Child Health and Human Development
9. Fertility Experiences Study/Joseph Stanford, MD/University of Utah
10. Longitudinal Investigation of Fertility and the Environment/Germaine Buck Louis, PhD/National Institute of Child Health and Human Development
11. National Birth Defects Prevention Study/Sarah C. Tinker, PhD/National Center on Birth Defects and Developmental Disabilities
12. National Survey of Family Growth/National Center for Health Statistics (<http://www.cdc.gov/nchs/nsfg/nsfg_questionnaires.htm>)
13. North Carolina Menopause Study/Donna Baird, PhD/National Institute of Environmental Health Sciences (<http://www.niehs.nih.gov/research/atniehs/labs/epi/studies/ncmno/question/>)
14. Ovarian Reserve after Cancer/Clarisa Gracia, MD/University of Pennsylvania
15. Pesticides, Endocrine Disruptors, Childhood Growth and Development/Mary S. Wolff, PhD/Mount Sinai School of Medicine
16. Right From The Start/Katherine Hartmann, PhD/Vanderbilt University
17. Study of Women’s Health Across the Nation/ National Institute on Aging, National Institute of Nursing Research, National Institutes of Health, Office of Research on Women’s Health, and the National Center for Complementary and Alternative Medicine (<http://www.swanstudy.org/docsharing/>)
18. Uterine Fibroid Study/Donna Baird, PhD/National Institute of Environmental Health Sciences (<http://www.niehs.nih.gov/research/atniehs/labs/epi/studies/ufs/question/>)
19. Women’s Health Initiative/National Institutes of Health, National Heart, Lung and Blood Institute (<https://biolincc.nhlbi.nih.gov/static/studies/whios/doc/whi/forms/whiforms.html>)
20. Women’s Interview Study of Health/National Cancer Institute (<http://dceg.cancer.gov/tools/design/questionnaires>)
21. Using Birth Certificates To Find Children Conceived With Infertility Treatment/Courtney Lynch, PhD & Germaine Buck Louis, PhD/National Institute of Child Health and Human Development
22. Fertility Information Research Study/H. Irene Su, MD/University of California, San Diego
23. Fertility and Parenthood After Cancer Study/Jessica R. Gorman, PhD/University of California, San Diego (Gorman JR, Su HI, Pierce JP, Roberts SC, Dominick SA, Malcarne VL. A multidimensional scale to measure the reproductive concerns of young adult female cancer survivors. Journal of Cancer Survivorship. 2014;8:218-28.)
24. Fertility and Reproduction in AYA Survivors Study/Jessica R. Gorman, PhD/ University of California, San Diego
25. Childhood Cancer Survivor Study/National Cancer Institute, ALSAC, St. Jude Children’s Research Hospital (<https://ccss.stjude.org/documents/questionnaires.html>)
